# T1 mapping of myocardium using inversion recovery cine at 3.0T

**DOI:** 10.1186/1532-429X-16-S1-P379

**Published:** 2014-01-16

**Authors:** Yanjie Zhu, Yinzhu Gao, Yiu-Cho Chung

**Affiliations:** 1 Shenzhen Institutes of Advanced Technology, Chinese Academy of Sciences, Shenzhen, Guangdong, China

## Background

T1 mapping is useful in the diagnosis of myocardial fibrosis [1]. It is commonly performed using MOLLI [2]. However, MOLLI and its variants are sensitive to arrhythmia, tissue T2 values, off-resonance, etc. and usually underestimates T1 [3]. We propose an arrhythmia insensitive myocardial T1 mapping technique at 3T that takes less than 6 seconds.

## Methods

The sequence, IR-rttfl, performs realtime turboflash acquisition after an inversion pulse to capture the recovery of inverted magnetization. It is immune to T1 unrelated issues (e.g., field inhomogeneity). Diastolic images (about 12) are selected for T1 calculation using [4]. A trigger delay (about 300-400 ms) is applied before the inversion pulse so that initial magnetization recovery was sampled in the first diastole. Parallel imaging (TGRAPPA rate 3) and asymmetric echo improve temporal resolution. The sequence was implemented on a 3T scanner (TIM Trio, Siemens). Its accuracy was tested in phantoms doped with gadolinium with known T1 values measured by spin echo. The technique was then evaluated in ten healthy volunteers (IRB approved with informed consent). T1 values from IR-rttfl and MOLLI were compared in four volunteers. Gadolinium contrast study was also performed on one healthy volunteer and one infarct patient. Imaging parameters: TR/TE = 2.3 ms/1.1 ms, flip angle = 5o, base matrix = 192, temporal resolution = 80-100 ms (subject dependent). The scan acquired 60 measurements in a breathhold (time < 6 s).

## Results

**Figure 1:**
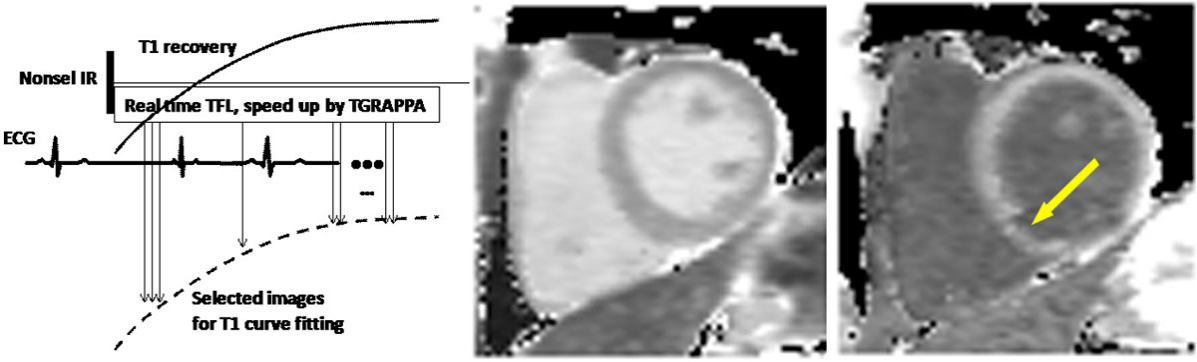
**The technique and typical T1 maps from the patient before and after contrast**.

Figure 1 shows IR-rttfl and a typical T1 map from the patient before and after contrast. In phantoms, IR-rttfl is more accurate than MOLLI (Table 1). In healthy volunteers, the myocardial T1s were 1254 ms ± 81 ms. In four of them, the T1 values from IR-rttfl and MOLLI were 1247 ms ± 83 ms and 1157 ms ± 68 ms respectively (deviation consistent with [3]). Partition coefficient in the healthy volunteer for IR-rttfl and MOLLI was 0.42 and 0.39 respectively. The T1 of the infarct 10 min after contrast injection was 278 ms and 352 ms for IR-rttfl and MOLLI respectively.

**Table 1:** Comparison of results of SE, IR-rttfl and MOLLI in phantom

	Spin Echo		IR- rttfl	MOLLI
**Phantom number**	**T2(ms)**	**T1(ms)**	**T1(ms)**	**T1(ms)**

1	346.7 ± 2.6	1505 ± 4	1518 ± 50	1403 ± 8

2	320.5 ± 0.9	1247 ± 3	1240 ± 35	1197 ± 8

3	284.0 ± 0.8	867 ± 3	870 ± 38	856 ± 7

4	260.6 ± 0.5	696 ± 2	693 ± 38	692 ± 7

5	177.0 ± 0.5	316 ± 1	313 ± 31	316 ± 12

6	160.8 ± 0.6	271 ± 1	252 ± 38	279 ± 7

## Conclusions

The arrhythmia insensitive IR-rttfl performs myocardial T1 mapping in a short breathhold. It is more accurate than MOLLI in phantoms. The results are comparable to MOLLI in healthy volunteers. Results from the initial contrast studies are promising.

## Funding

Guangdong innovation team, Sichun province collaboration project.
